# Relationship between Dizziness and the Core Vestibular Projection Injury in Patients with Mild Traumatic Brain Injury

**DOI:** 10.3390/diagnostics11112070

**Published:** 2021-11-09

**Authors:** Sung-Ho Jang, Chang-Hoon Bae, Jae-Woon Kim, Hyeok-Gyu Kwon

**Affiliations:** 1 Department of Physical Medicine and Rehabilitation, College of Medicine, Yeungnam University, Daegu 42415, Korea; strokerehab@hanmail.net; 2 Department of Otorhinolaryngology-Head and Neck Surgery, College of Medicine, Yeungnam University, Daegu 42415, Korea; baich@med.yu.ac.kr; 3 Department of Radiology, College of Medicine, Yeungnam University, Daegu 42415, Korea; sungho1999@ynu.ac.kr; 4 Department of Physical Therapy, College of Health Science, Eulji University, Sungnam-si 13135, Korea

**Keywords:** dizziness, core vestibular projection, vestibular nuclei, parieto-insular vestibular cortex, mild traumatic brain injury, diffusion tensor tractography

## Abstract

Some studies have reported that a core vestibular projection (CVP) injury is associated with dizziness following a brain injury using diffusion tensor tractography (DTT). On the other hand, there has been no DTT study on dizziness caused by a CVP injury in patients with mild traumatic brain injury (TBI). In this study, DTT was used to examine the relationship between dizziness and CVP injury in patients with mild TBI. Forty-three patients with mild TBI and twenty-nine normal subjects were recruited. The patients were classified into two groups based on the dizziness score: group A, patients with a dizziness score less than 2 on the sub-item score for dizziness in the Rivermead Post-concussion Symptoms Questionnaire; group B, patients with a dizziness score above 2. The tract volume (TV) in group B was significantly lower than group A and the control group (*p* < 0.05). By contrast, the TV in group A was similar to the control group (*p* > 0.05). Regarding the correlation, the dizziness score of all patients showed a strong negative correlation with the TV of the CVP (*r* = −0.711, *p* < 0.05). DTT revealed the CVP injury in patients with dizziness after mild TBI. In addition, the severity of dizziness of these patients was closely related to the injury severity of the CVP.

## 1. Introduction

Dizziness is an umbrella term that includes a range of symptoms, such as vertigo, disorientation, and light-headedness [1,2]. The condition occurs because of an impairment of spatial orientation and motion perception [3]. Regarding the pathophysiological mechanism of dizziness, two main causes are known [4,5]: (1) injury to the vestibular system (benign paroxysmal positional vertigo (BPPV) and labyrinthine concussion), and (2) injury to the non-vestibular system (central system injury and psychological factors). Although the most common cause of dizziness is BPPV, a central vestibular system injury after a brain injury is also a common cause [6]. The central vestibular system has a unique role in sensorimotor control and perception. It consists of the vestibular nuclei (VN), cerebellum, thalamus, and vertebral cortex, particularly the parieto-insular vestibular cortex (PIVC), which is a core region of the vestibular cortex [7,8]. Among these brain regions, the PIVC is involved in processing motion perception and spatial orientation, in particular the correlation with head motion in space, twisting the neck, and the motion of a visual target [7,9,10,11]. The pathway between the PIVC and VN is called the core vestibular projection (CVP). Hence, injury to the CVP is associated with dizziness [12,13].

A traumatic brain injury (TBI) causes focal and diffuse brain injury and is classified into three types (mild, moderate, and severe) according to the severity; mild TBI has been reported in up to 85% of cases of TBI [14,15,16]. The majority of cases of mild TBI presented at least one clinical symptom, such as headache, dizziness, anxiety, depression, and fatigue [17,18,19,20]. Among these clinical symptoms, dizziness is the most common clinical symptom and has been reported in up to 80% of cases following a mild TBI [1,21,22]. Therefore, it is important to determine the precise pathophysiological mechanism of dizziness for management in patients with mild TBI. On the other hand, little is known about this topic in patients with mild TBI.

Precise estimations of CVP have been limited because it has not clearly discriminated from the adjacent neural structures on conventional brain MRI. By contrast, diffusion tensor tractography (DTT), which is derived from diffusion tensor imaging (DTI), can detect invisible or micro-injuries to the brain by measuring the diffusion of water molecules in the white matter and estimating the CVP by a three-dimensional reconstruction [23,24,25]. Meanwhile, edema, which is a hallmark of TBI, should be controlled before applying DTI. The glymphatic system is a waste clearance as a brain-wide network of perivascular pathways. Acuapoin-4, a plasma membrane channel, affects the glymphatic function and a key role in the formation and clearance of cerebral edema [26,27,28]. Recent studies demonstrated that targeting acuapoin-4 using trifluoperazine was a viable treatment for edema [29,30]. Some studies have reported that a CVP injury is associated with dizziness after ischemic and hemorrhagic stroke using DTT [31,32,33]. However, there are no reports of DTT studies on dizziness caused by an injury to the CVP in patients with mild TBI. In the current study, DTT was used to examine the relationship between dizziness and a CVP injury in patients with mild TBI.

## 2. Methods

### 2.1. Subjects

Forty-three patients (male: 15, female: 28, mean age: 39.3 ± 9.0 years, range: 21–49 years) with mild TBI and twenty-nine age- and sex-matched healthy control subjects (male: 11, female: 18, mean age: 38.5 ± 7.3 years, range: 23–49 years) with no prior history of neurological, physical, or psychiatric illness were recruited for this study (Table 1). The following inclusion criteria were used to recruit the patients: (1) loss of consciousness (LOC) for <30 min, posttraumatic amnesia (PTA) for ≤24 h, and initial Glasgow Coma Scale (GCS) score of 13–15 [15];_ENREF_22 (2) no specific lesion observed on brain MRI (T1-weighted, T2-weighted, and fluid-attenuated inversion recovery images); (3) more than one month elapsed since the onset of TBI; (4) age range: 20–50 years; (5) presence of dizziness that excluded the other causes except for the brain as confirmed by an otolaryngologist. All healthy subjects understood the purpose of the study and provided written, informed consent before starting this study. The patients’ consent could not be obtained because this study was conducted retrospectively. The study protocol without the patients’ consent was approved by the Institutional Review Board of a local university hospital.

### 2.2. Clinical Evaluation

Dizziness in the sub-item score for the dizziness of Rivermead Post-concussion Symptoms Questionnaire (RPQ) was used during DTI scanning. The sub-item scores of dizziness of the RPQ were as follows: full score = 4; 0 points = not experienced at all; 1 point = not a problem; 2 points = mild problem; 3 points = moderate problem; 4 points = severe problem [34]. The patients were classified according to two groups based on the dizziness score of the RPQ: group A, patients with a dizziness score of the RPQ less than 2 (<2); group B patients with a dizziness score of the RPQ above 2 (≥2). Table 1 lists the demographic and clinical data for the three groups.

### 2.3. Diffusion Tensor Tractography

DTI data were acquired an average of 6.4 ± 7.9 months after the onset of head trauma using a six-channel head coil on a 1.5 T Philips Gyroscan Intera with 32 non-collinear diffusion sensitizing gradients (Philips, Ltd., Best, The Netherlands). The imaging parameters were as follows: acquisition matrix = 96 × 96; reconstructed to matrix = 192 × 192; field of view = 240 × 240 mm^2^; repetition time = 10,398 ms; echo time = 72 ms; parallel imaging reduction factor (SENSE factor) = 2; echo-planar imaging factor = 59; b = 1000 s/mm^2^; a slice thickness of 2.5 mm. The head motion effects and image distortions due to the eddy current were corrected by applying affine multi-scale two-dimensional registration using the Functional Magnetic Resonance Imaging of the Brain (FMRIB) Software Library (FSL; www.fmrib.ox.ac.uk/fsl, accessed on 3 May 2021) [35]. One analyzer (Kwon HG) performed fiber tracking using probabilistic tractography, which was applied in the default tractography option (5000 streamline samples, 0.5 mm step lengths, curvature thresholds = 0.2) of the Oxford Centre for FMRIB Software Library [35,36]. The CVP was reconstructed from a seed region of interest at the VN corresponding to Schwalbe’s and Deiters’ nuclei at the pons level [31]. The target region of interest was positioned at the PIVC [31]. The core vestibular projection passes from the vestibular nuclei at the level of the pons to the PIVC via the posterolateral thalamus [9]. The values of fractional anisotropy (FA), mean diffusivity (MD), and tract volume (TV) were determined for each subject.

### 2.4. Statistical Analysis

Statistical analyses were performed using SPSS software (v. 25.0; SPSS, Chicago, IL, USA). One-way analysis of variance (ANOVA) was performed to determine differences in FA, MD, and TV values between the three groups. When a significant difference was detected among the three groups, a Bonferroni post hoc test was performed to determine the differences in FA, MD, and TV values among the three groups. Using Pearson correlation, the dizziness score in the Rivermead Post-concussion Symptoms Questionnaire was used to determine the correlation with the FA, MD, and TV values. The correlation coefficient (*r*) indicates the relative strength (0.1–0.3: weak, 0.3–0.5: moderate, >0.5: strong) and direction (+, −) of a linear relationship between two values [37]. A *p*-value < 0.05 was considered significant.

## 3. Results

**Figure 1:**
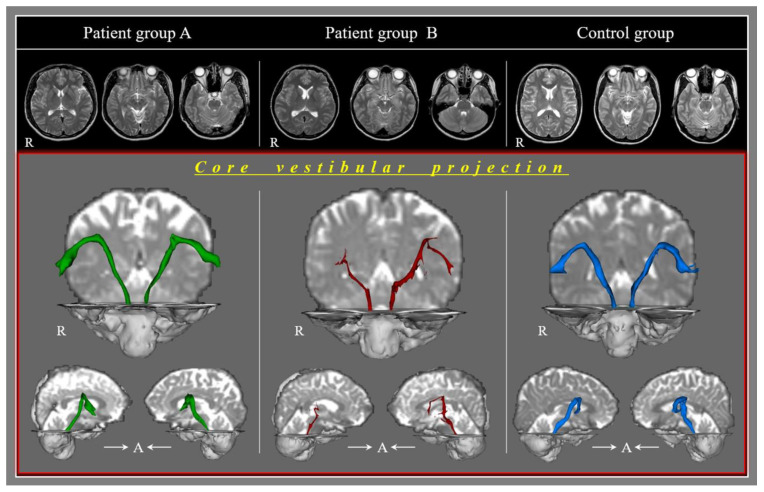
Results of diffusion tensor tractography of the core vestibular projection according to the three groups.

Table 2 lists the DTT parameters for the CVP and the correlations between the dizziness score and DTT parameters of the CVP (Table 2). Group B showed a significantly lower TV than group A and the control group (*p* < 0.05) (Figure 1). By contrast, group A showed a similar TV to the control group (*p* > 0.05). The FA and MD values were similar in the three groups (*p* > 0.05).

The dizziness score of all patients showed a strong negative correlation with the TV of the CVP (*r* = −0.711) (*p* < 0.05). On the other hand, the correlation between the dizziness score and the FA and MD values of the CVP was weak (*p* > 0.05).

## 4. Discussion

This study investigated the relationship between dizziness and the CVP state in patients with mild TBI. The results are summarized as follows. (1) the TV of the CVP in group B was lower than that of group A and the control group. However, no significant difference in any DTT parameters was observed and group B was the only group that showed dizziness. We thought that group B was more injured by the CVP caused by traumatic axonal injury than both group A and the control group. (2) The dizziness score showed a strong negative correlation with only the TV of the CVP. A quantitative assessment of the state of a neural tract is possible by the DTT parameters, and FA, MD, and TV are used widely [38,39]. The FA value indicates the degree of directionality of water diffusion. Consequently, it reflects the fiber density, axonal diameter, and myelination of the white matter, whereas the MD value considers the magnitude of water diffusion [38]. In contrast, the TV value represents the volume of the neural tract by counting the number of voxels [39]. Hence, the decrease in the TV value of the patients (group B) who presented with dizziness after mild TBI indicated an injury to the CVP. Because the patients in the present study did not show any specific lesions on conventional MRI, a traumatic axonal injury, which is defined as an injury of axons due to indirect shearing forces during acceleration, deceleration, and rotation of the brain, or direct head trauma appeared to be a plausible pathogenic mechanism for a CVP injury [40,41,42]. The other result showing that the dizziness score was strongly negatively correlated with the TV of the CVP suggests that the severity of dizziness was closely related to the injury severity of the CVP.

Many studies reported dizziness after a mild TBI [1,21,22,43,44,45]. By contrast, little is known about the pathophysiological mechanisms related to the central system origin [46,47]. In 2006, Endo et al. reported that the dizziness of patients with a whiplash injury was caused by asymmetry of the vertebrobasilar artery, which often affects the brainstem and causes cerebellar ischemia [46]. Hong et al. (2010) reported that injuries to the vestibular nuclei and nerve caused dizziness in a stroke patient [47]. Regarding the studies based on DTT for the CVP, some studies have reported CVP injury in stroke [31,32,33]. Yeo et al. (2017) attributed the vertigo of 19 patients with middle cerebral artery infarction to an injury to the CVP [31]. They reported that the decrement of the TV of the CVP was caused by lesions due to the middle cerebral artery infarction. In 2018, Yeo et al. showed that the CVP injury was associated with dizziness after a lateral medullary infarction including the VN [32]. In 2020, Kwon et al. stated that a discontinuation of the CVP at the basal ganglia level was related to dizziness in a patient with intracerebral hemorrhage [33]. The CVP originated from the VN, ascended at the level of the internal capsule via the posterolateral thalamus, and terminated on the PIVC. Because both the VN and the PIVC are associated with dizziness, the above studies suggested that the CVP injury was associated with dizziness and coincided with the present results. Meanwhile, Yeo et al. (2020) reported that the TV of the CVP was associated with age [48]. Thus, we recruited the patients who were younger than 50 years old in this study. This is the first study to demonstrate an injury of the CVP in patients with mild TBI to the best of the authors’ knowledge.

This study is expected to benefit patients exhibiting dizziness after mild TBI because a brain lesion in mild TBI is difficult to detect using conventional MRI. Nevertheless, several limitations should be considered. First, detailed information on dizziness could not be acquired. Second, DTT could lead to both false-positive and negative results throughout the white matter of the brain because of complex fiber configurations, such as crossing or kissing fiber and partial volume effects [49]. Therefore, further histopathological studies to confirm traumatic axonal injury in an animal model or autopsy would be necessary [40,41,42]. In addition, regarding the molecular insight, one would need to use a humanized system allowing real-time monitoring of brain penetration and dynamic volume change during TBI [50].

In conclusion, DTT was used to examine a CVP injury in patients with dizziness after mild TBI. In addition, the severity of dizziness of these patients was closely related to the injury severity of the CVP. Therefore, injured CVP could be involved the dizziness following the mild TBI. These results suggest that DTI could be useful for detecting a CVP injury that may not be detected on conventional brain MRI in patients with mild TBI. In addition, measurement of the CVP could be applied to the evaluation of the dizziness following the brain injury.

## Figures and Tables

**Table 1:** Demographic and clinical data of the patient and control groups.

	Group A	Group B	Control Group
Sex (male:female)	7:8	8:20	11:18
Mean age, years	38.7 (10.6)	39.5 (8.5)	38.5 (7.3)
LOC, minutes	0.8 (1.7)	3.8 (7.7)	-
PTA, minutes	4.6 (10.8)	10.2 (16.9)	-
GCS score	15.0 (0.0)	15.0 (0.0)	-
Mean duration to DTI, months	5.2 (4.9)	7.1 (9.3)	-
Dizziness score	0.8 (0.4)	3.0 (0.7)	-

Values represent mean (± standard deviation), LOC: loss of consciousness, PTA: posttraumatic amnesia, GCS: Glasgow Coma Scale, DTI: diffusion tensor imaging.

**Table 2:** Diffusion tensor tractography parameter values of the core vestibular projection of the patient and control groups.

	Group A	Group B	Control Group
Fractional anisotropy	0.44	0.43	0.43
	(0.03)	(0.07)	(0.03)
Mean diffusivity	0.83	0.83	0.82
	(0.05)	(0.06)	(0.05)
Tract volume	595.53	345.59 †	554.21
	(151.51)	(240.22)	(198.15)
Correlation between the dizziness score and diffusion tensor tractography parameters			
	Fractional anisotropy	Mean diffusivity	Tract volume
Core vestibular projection	0.037	0.091	−0.711 *

Values represent the mean (±standard deviation). †: Significant differences between group A and group B and between group B and control group, and no significant difference between group A and control group. * Significant negative correlation between the dizziness score and diffusion tensor tractography parameters, *p* < 0.05.
